# Making spectral shape measurements in inverse Compton scattering a tool for advanced diagnostic applications

**DOI:** 10.1038/s41598-018-19546-0

**Published:** 2018-01-23

**Authors:** J. M. Krämer, A. Jochmann, M. Budde, M. Bussmann, J. P. Couperus, T. E. Cowan, A. Debus, A. Köhler, M. Kuntzsch, A. Laso García, U. Lehnert, P. Michel, R. Pausch, O. Zarini, U. Schramm, A. Irman

**Affiliations:** 1 Institute of Radiation Physics, Helmholtz-Zentrum Dresden - Rossendorf, Bautzner Landstrasse 400, 01328 Dresden, Germany; 2 Technische Universität Dresden, 01062 Dresden, Germany; 3 Danfysik A/S, Gregersensvej 8, 2630 Taastrup, Denmark; 4 Present Address: National Energetics Inc., 4616 W Howard Ln, Austin, TX 78728 USA

## Abstract

Interaction of relativistic electron beams with high power lasers can both serve as a secondary light source and as a novel diagnostic tool for various beam parameters. For both applications, it is important to understand the dynamics of the inverse Compton scattering mechanism and the dependence of the scattered light’s spectral properties on the interacting laser and electron beam parameters. Measurements are easily misinterpreted due to the complex interplay of the interaction parameters. Here we report the potential of inverse Compton scattering as an advanced diagnostic tool by investigating two of the most influential interaction parameters, namely the laser intensity and the electron beam emittance. Established scaling laws for the spectral bandwidth and redshift of the mean scattered photon energy are refined. This allows for a quantitatively well matching prediction of the spectral shape. Driving the interaction to a nonlinear regime, we spectrally resolve the rise of higher harmonic radiation with increasing laser intensity. Unprecedented agreement with 3D radiation simulations is found, showing the good control and characterization of the interaction. The findings advance the interpretation of inverse Compton scattering measurements into a diagnostic tool for electron beams from laser plasma acceleration.

## Introduction

Inverse Compton scattering (ICS) or Thomson backscattering of intense laser pulses off relativistic electrons attracts increasing attention because it can generate ultra-short and narrow bandwidth light pulses, tunable from the extreme ultraviolet (EUV) to the *γ*-ray regime, while requiring far lower electron energies than conventional light sources based on magnetic insertion devices^1,2^. It offers a novel research tool in a broad range of fundamental and applied science^3,4^, e.g. medical X-ray fluorescence imaging or computed tomography^5,6^, ultra-fast X-ray diffraction in high-energy-density physics^7–9^ or ultra-high-intensity-laser matter interactions^10^, photo-nuclear applications and radiography^11–15^. The narrow but finite bandwidth of this light source is of particular interest for these applications, which are typically complex and/or destructive processes, enabling for example the detection of a sharp resonance or absorption edge in a certain spectral range within a single exposure while maintaining a low background signal.

While conventional accelerator driven Compton light sources^16–21^ are well suited for high repetition rate and accordingly high average photon flux, competitive high peak brilliance in a single exposure is difficult to achieve. A promising candidate with unique properties for these applications is a laser wakefield accelerated (LWFA) electron beam^22–24^. The ultra-short pulse duration (fs) and accordingly ultra-high peak current, the wide energy tuning range and the compact design are some of the benefits over conventional accelerator technology^25^. Furthermore, electron and scattering laser beams are inherently synchronized and suffer less from timing jitter, since they are generated by the same laser oscillator. Although remarkable progress has been made in controlling the LWFA process, in reaching high energies (GeV)^26–28^ and high peak currents (tens of kA)^29–31^, operation of an all optical ICS light source is not feasible for applications yet. Further optimization of electron beam parameters like energy spread and divergence is still required and stability of operation has to improve. The nonlinear nature of LWFA makes this effort challenging. Finally, extracting the beam out of the plasma and transporting it while maintaining its high initial quality is subject to ongoing investigations^4,32–36^. Especially for the latter, a good single shot diagnostic in this difficult to access region is crucial for understanding the beam dynamics directly at the exit of the plasma accelerator. Such a tool is currently not available. Conventional diagnostic tools fail due to spatial constraints, disruptive electromagnetic pulses from the laser plasma interaction, or are not suitable for the LWFA typical parameter range. In this paper we show that ICS can provide a solution for this diagnostic task, in addition to its prospect as a secondary light source, since the interaction is sensitive to relevant electron beam parameters.

Advanced beam diagnostic based on ICS has been suggested and applied by other groups, e.g. to determine the electron energy^37^ or transverse beam parameters^21,38,39^. Such tools are non-invasive and therefore suitable for online monitoring as well as capable of single shot measurements. A further advantage is that the measurement position can easily be adjusted, e.g. to track the transverse emittance after extraction out of the plasma. However, the complex interplay of several interaction parameters and the lack of alternative beam diagnostics as a reference make calibration of this novel diagnostic tool a delicate endeavor in an LWFA setup. Thus as the main focus of this paper, the influence of the laser and electron beam parameters on the scattered photon spectral shape is carefully investigated at a conventional accelerator, where conventional beam diagnostics are available and independent parameter tuning is possible. Besides the advanced diagnostic applications, the presented work can as well be important for secondary light source design and characterization. Hence, we will also report on source parameters, e.g., the photon flux and peak brilliance with the reservation of not having optimized the experiments for best light source performance.

Previous experimental work characterizing ICS sources at conventional accelerators include the study of the angularly resolved spectrum by Jochmann *et al*.^21^. Furthermore, Babzien *et al*.^40^ and Sakai *et al*.^41^ observed redshifting of the spectrum with laser intensity as well as higher harmonic radiation. For the use of Compton scattering as beam diagnostic, a quantitative description of the influence of various beam and interaction parameters is mandatory. However, comparison of measured ICS spectra to full 3D simulations with numerous free fitting parameters are too time consuming to be feasible as an online diagnostic. Accordingly, mostly theoretical models and simulations with strongly simplified models are used for quick analysis so far. Here, we derived and confirmed the quantitative dependence of the on-axis spectrum in terms of mean scattered energy and spectral bandwidth on the electron beam divergence and laser intensity based on thorough experimental investigation. In the measurements, the parameter space comparable to LWFA based ICS sources was covered (*a*_0_ = 0.05 to 1.6 and electron beam divergence 0.1 to 11.8 mrad). The findings are well supported by 3D radiation simulations, which show agreement with the experimental data at an unprecedented precision. We anticipate that this study will not only impact on the future design of high brightness ICS light sources but also advance innovative electron beam diagnostics particularly at difficult environment for example electron storage rings and LWFA.

## Results

### Kinematics of inverse Compton scattering

For moderate laser intensities and electron beam energies presented in this article, quantum effects in inverse Compton scattering can be neglected. The energy of the scattered photons is much smaller than the electron energy, any effect of the scattering process on the electron beam is below the detection limit. This low energy limit can accordingly be treated classically and is also referred to as Thomson scattering. Here, the laser field is the equivalent to an optical undulator (linear Thomson scattering) or wiggler (nonlinear Thomson scattering). Due to the sub-micrometer period of the optical wavelength, the same scattered photon energy can be obtained at two orders of magnitude smaller electron energy as compared to electrons passing through magnetic undulators. The laser strength parameter *a*_0_ corresponds to the undulator strength parameter^42^. For ${a}_{0}\ll 1$ electrons oscillate transversely, along the polarization plane of the laser, thereby radiating within a narrow bandwidth, boosted and collimated in the electron beam forward direction. With increasing laser strength $({a}_{0}\gtrsim 1)$, the influence of the laser’s magnetic field component becomes comparable to that of the electric field and forces the electron on a figure of eight motion in the electron rest-frame instead. The polarization of the emitted light is determined by the laser polarization, enabling easy tuning as required for certain experiments. In a semi-classical picture, the fundamental radiation of the interaction for one electron can be described by the following equation^43^:

1 $${\omega }_{{\rm{sc}}}=\frac{2{\gamma }^{2}(1-\,\cos \,\phi )}{1+\frac{{a}_{0}^{2}}{2}+{(\gamma \theta )}^{2}}{\omega }_{0}.$$

**Figure 1:**
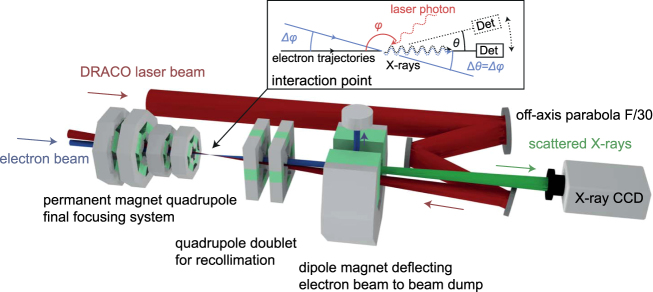
Schematic drawing of the interaction geometry. The electron beam (blue) is focused to the interaction point with a magnetic final focusing system. After interaction it is collimated again and deflected to a beam dump with a dipole magnet. The laser (red) is focused with an off-axis parabola (F/30). The interaction angle is close to head-on with a small angle of 22 mrad to have a clear path for the X-rays (green). The X-rays are detected with a CCD camera. Angles at the interaction point are illustrated in the schematic drawing of the interaction.

Here, each laser photon with the angular frequency *ω*_0_ scatters off a relativistic electron. Their energy is Doppler-upshifted depending on the electron energy *γm*_e_*c*^2^, the laser strength *a*_0_, the scattering angle *φ* and the observation angle *θ* (see Fig. 1).

The maximum amplification factor of the scattered photon energy is 4*γ*^2^, achieved at the electron axis for a head-on collision. Symmetric broadening of the scattered photon energy induced by the intrinsic laser spectral bandwidth and the electron beam energy spread defines the lower limit of the spectral bandwidth Δ*ω*_sc_/*ω*_sc_^13,44^. Ultra-short pulse laser systems have an intrinsic broadband spectrum, resulting in a significant broadening effect (∝Δ*ω*_0_/*ω*_0_) of up to a few percent. For conventional accelerators, the spectral broadening effect caused by the electron beam energy spread is typically negligible. For laser wakefield based electron sources, which possess typically a few percent relative energy spread, this contribution (∝2Δ*γ*/*γ*) can be on a several percent level, similar to the laser intrinsic bandwidth. According to Eq. 1, the scattered frequency drops with the observation angle *θ*^21,43,45^. The effect of the electron beam emittance or divergence and laser intensity is strongly coupled to the resulting angular and spectral shape. At the electron axis, both parameters lead to an asymmetric broadening and redshift of the mean or peak energy. This correlation to the beam parameters can be utilized for diagnostic purposes by measuring the ICS spectrum. However, scaling laws for these quantities found in literature^13,44^ do not include detailed geometrical considerations, i.e. shape and size of both beams strongly affect the scattered spectrum. Technically speaking, Eq. 1 only describes the spectrum of a single electron-photon collision, whereas measured spectra are averaged over the ensemble of collisions. Attempting to verify the scaling laws, we accordingly found significant disagreement to the experiment. We show how this can be overcome by properly taking the interaction geometry, hence volume, into account. As a consequence, the *effective divergence* of the electron beam and the *effective laser strength parameter* are defined in the following as more suitable quantities for the description of the spectral dependence. Similar concepts have been discussed in the different context of luminosity of colliding beams^46,47^.

### Effective quantities of the scattering process

The scattering probability and thus the contribution to the fundamental of the spectrum (number of scattered photons *N*_*ph*_) is not only proportional to the electron bunch charge *Q* but also to the laser intensity *I*, ${N}_{{\rm{ph}}}\propto I\times Q\propto {a}_{0}^{2}\times Q$. As the electrons sweep through the laser pulse during the interaction, they oscillate in the local field of the laser. The envelope of this field can be approximated by a 3D Gaussian function *a*_0_ · *g*(*x*, *y*, *ct*) with *a*_0_ as the peak value for focused laser beams. For the correlation of the spectral broadening effects to the laser strength, the *effective laser strength a*_0,eff_ is defined as a weighted laser strength. Here, the number of scattered photons in the fundamental is chosen as the weighting function

2 $${a}_{\mathrm{0,}{\rm{eff}}}=\frac{\int dV\,{N}_{{\rm{ph}}}(x,y,ct)\,{a}_{0}\,g(x,y,ct)}{\int dV\,{N}_{{\rm{ph}}}(x,y,ct)}={a}_{0}\,\frac{\int dV\,q(x,y)\,g{(x,y,ct)}^{3}}{\int dV\,q(x,y)\,g{(x,y,ct)}^{2}},$$

with the projected electron charge density function *q*(*x*,*y*) (transverse profile). By this means, the effective laser strength contributing to the bandwidth of the Thomson spectrum is smaller than the (peak) laser strength parameter. This definition considers both the shape of the laser and the interaction volume. By modifying the electron beam phase space distribution, it is also possible to account for the specifics of other experiments or geometries, e.g. potential transverse displacement of the electron beam for interaction angles other than 180° or the hourglass effect (change in beam size during interaction) for electron beams with long pulse duration compared to their beta-function. Both effects are not relevant for the beam parameters used in this work. Depending on the transverse distribution and size of the electron beam, the spatial overlap of both beams is affected and the effective laser strength is reduced to a value between 54.4% (for electron beam size ${r}_{e}\,\gg$ laser size *r*_*l*_) and 81.6% of the peak value (for ${r}_{e}\ll {r}_{l}$), taking Gaussian distributed beams. An electron beam matching the laser beam size leads to an effective laser strength of 61.2% of the peak value.

Same considerations apply to the effective electron beam divergence *σ*_*θ*,eff_. It is defined as the weighted mean of the scattering angle ${\rm{\Delta }}{\phi }_{i}={\rm{\Delta }}{\theta }_{i}=\sqrt{{x^{\prime} }_{{\rm{i}}}^{2}+{y^{\prime} }_{{\rm{i}}}^{2}}$ in 2D caused by the electron beam divergence (see Fig. 1) and reads as

3 $${\sigma }_{\theta ,{\rm{eff}}}=\frac{{\sum }_{{\rm{i}}\mathrm{=1}}^{{N}_{{\rm{e}}}}({a}_{0}^{2}\,g{({x}_{{\rm{i}}},{y}_{{\rm{i}}})}^{2}\cdot \sqrt{{x^{\prime} }_{{\rm{i}}}^{2}+{y^{\prime} }_{{\rm{i}}}^{2}})}{{\sum }_{{\rm{i}}\mathrm{=1}}^{{N}_{{\rm{e}}}}{a}_{0}^{2}\,g{({x}_{{\rm{i}}},{y}_{{\rm{i}}})}^{2}},$$

where *N*_e_ is the number of electrons and $({x}_{{\rm{i}}},{x^{\prime} }_{{\rm{i}}}=d{x}_{{\rm{i}}}/dz,{y}_{{\rm{i}}},{y^{\prime} }_{{\rm{i}}}=d{y}_{{\rm{i}}}/dz)$ are the phase space coordinates for one electron. Please note that the geometric divergence of an electron beam is typically defined differently as the root-mean-square (RMS) of the 1D angles in the horizontal and vertical plane. Depending on the phase space distribution and the spatio-temporal overlap, the effective divergence can be smaller than the geometric divergence, e.g. in case of a diverging beam and *r*_e_ > *r*_l_. For ICS based beam diagnostic of electron beams from LWFA, this requires a good control of the spatio-temporal overlap as well as *r*_l_ > *r*_e_ at the interaction point in order not to underestimate the divergence.

As the result and from Eq. 1, the redshift of the fundamental’s mean scattered energy on axis for relativistic electrons and a head-on geometry reads as

4 $$\langle \hslash {\omega }_{{\rm{sc}}}\rangle =\frac{4{\gamma }^{2}\hslash {\omega }_{0}}{1+\frac{{({a}_{\mathrm{0,}{\rm{eff}}})}^{2}}{2}+{(\gamma {\sigma }_{\theta ,{\rm{eff}}})}^{2}}\mathrm{.}$$

In terms of spectral broadening the contributions are

5 $${(\frac{{\rm{\Delta }}\omega }{\omega })}_{{a}_{0}}\simeq \frac{0.88{a}_{\mathrm{0,}{\rm{eff}}}^{2}}{2+{a}_{\mathrm{0,}{\rm{eff}}}^{2}}$$

and

6 $${(\frac{{\rm{\Delta }}\omega }{\omega })}_{{\sigma }_{\theta }}\simeq \frac{1.05{(\gamma {\sigma }_{\theta ,{\rm{eff}}})}^{2}}{1+{(\gamma {\sigma }_{\theta ,{\rm{eff}}})}^{2}}\mathrm{.}$$

The derivation of the equations is given in Supplementary Note 2 and 3. For Eqs 5 and 6 radially symmetric Gaussian beams are assumed. Eq. 5 additionally assumes the spatial overlap to be ${r}_{l}\ll {r}_{e}$. In other cases a different correction factor would apply, e.g. 0.5 for ${r}_{l}\gg {r}_{e}$ or 0.72 for *r*_*l*_ = *r*_*e*_.

For most LWFA based ICS sources (*a*_0,eff_ ≥ 0.5 and *γσ*_*θ*,eff_ > 0.5), the broadening effect due to the laser intensity and the electron beam divergence will thus dominate the spectrum and exceed the symmetric broadening caused by the intrinsic laser bandwidth and the electron beam energy spread. In total, the bandwidth of the ICS spectrum sums up to

7 $$\frac{{\rm{\Delta }}{\omega }_{sc}}{{\omega }_{sc}}\approx \sqrt{{(\frac{{\rm{\Delta }}{\omega }_{0}}{{\omega }_{0}})}^{2}+{(\frac{2{\rm{\Delta }}\gamma }{\gamma })}^{2}+{(\frac{{\rm{\Delta }}\omega }{\omega })}_{{\sigma }_{\theta }}^{2}+{(\frac{{\rm{\Delta }}\omega }{\omega })}_{{a}_{0}}^{2}}.$$

Stating the obvious, isolated treatment of broadening effects can lead to wrong conclusions. Specifically in the LWFA case, the asymmetric broadening cannot be measured solely for the deduction of the electron beam divergence without considering the laser intensity or vice versa.

### Influence of the electron beam emittance

In this work, electron beams are provided by the ELBE superconducting linear accelerator and focused by a permanent magnet based final focusing system^48^ (FFS) onto the interaction point with the counter-propagating DRACO laser beam, see Fig. 1. The emittance of the electron beam was measured at the interaction point with a 3D wirescan method (see Supplementary Material), probing the beam waist in the vicinity of the focus. Based on these measurements the 4D (transverse) phase space was synthesized assuming Gaussian distributions. Later in the analysis, this phase space information is used for the derivation of the effective divergence and serves as a model of the electron beam for 3D radiation simulations.

The definition of the normalized RMS emittance for a particle distribution is

8 $${\varepsilon }_{N}=\beta \gamma \sqrt{\langle {x}^{2}\rangle \langle {x^{\prime} }^{2}\rangle -{\langle xx^{\prime} \rangle }^{2}},$$

assuming a small energy spread and *β* = *v*/*c* being the normalized electron velocity. In terms of macroscopic quantities, the emittance is defined as

9 $${\varepsilon }_{N}=\beta \gamma {\sigma }_{x}{\sigma }_{\theta },$$

with the RMS beam size *σ*_*x*_ and the RMS divergence *σ*_*θ*_. Both are measured at a focus position, where the transverse phase space is uncorrelated.

**Figure 2:**
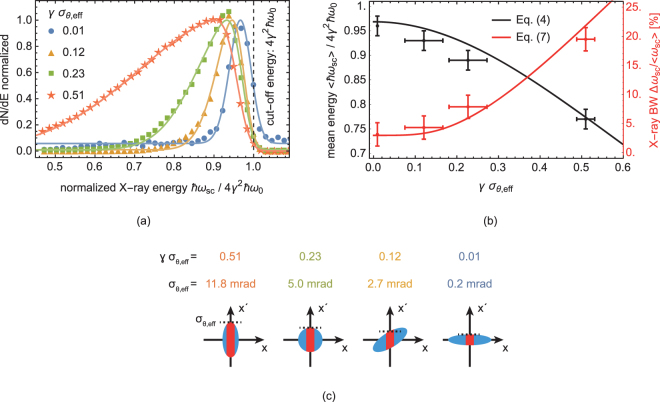
Broadening and redshift of the ICS spectra with increasing effective divergence of the electron beam and 4*γ*^2^*ħω*_0_ = 12.9 keV. (**a**) Measured ICS spectra (dots) with fitted skew normal distributions (solid lines) to derive mean energy and bandwidth. The normalization of the plot causes the cut-off energy for *γσ*_*θ*,eff_ = 0.01 to appear blueshifted, since the absolute flux was lower than in the other measurements by a factor of about 50. (see Supplementary Figure 2 for spectra with absolute photon numbers). (**b**) Dependence of mean energy and bandwidth derived from (**a**) on the effective divergence and comparison to Eqs 4 and 7. Error bars represent the measurement uncertainty of the electron beam divergence and the X-ray detector resolution. (**c**) Sketch (not to scale) of the electron beam phase space (blue) and the overlap with the laser (red) for the spectra in (**a**).

The effective divergence was varied experimentally by changing the strength of the FFS. For a given emittance, stronger focusing to the interaction point results in a larger divergence. Since the laser focal spot size was smaller than the focal spot of the electron beam in the presented work, only a part of the electron beam was interacting and thus contributing to the effective divergence according to Eq. 3. In order to investigate the influence of the effective divergence to the spectral broadening of the ICS X-ray beam, a set of four measurements was performed by varying the effective divergence in the following way, see Fig. 2(c). Two data points (5.0 mrad and 2.7 mrad) were taken for the same focusing strength of the FFS but different spatial overlap. The reduction to 2.7 mrad was achieved by shifting the entire FFS and thus the focus position upstream the electron beam axis by 10 mm. Accordingly, only a part of the so obtained correlated phase space distribution (tilted phase space ellipse) was interacting with the laser. For this specific case, the flux at the peak energy was not affected. Only the low energy tail of the spectrum was cut away, which corresponds to the outer fraction of the electron phase space. In the third measurement (11.8 mrad) the strength of the FFS was increased. The electron beam was focused to the smallest spot resulting in a better overlap with the laser beam and generating a higher flux and larger bandwidth X-ray beam. The smallest divergence was obtained by removing the FFS from the setup creating a millimeter size electron beam (>50 × *r*_*l*_). The effective divergence in this case was smaller than 0.2 mrad.

Figure 2(a) shows the ICS spectra, measured for the *γσ*_*θ*,eff_ of 0.01, 0.12, 0.23 and 0.51. The asymmetric broadening effect can clearly be seen by the bandwidth increase and the redshift of the spectra with increasing effective divergence. For a quantitative analysis, skew normal distributions are fitted to the spectra. The redshift is determined from the mean energy of the fitted distribution. The relative bandwidth is defined as its standard deviation divided by the mean energy. This method is preferred over direct statistics on the data since parts of the low energy tail could not be resolved for the measurements with the largest effective divergence. Radiation below 5.5 keV (≈0.45 × 4*γ*^2^*ħω*_0_) was blocked by absorption within an air gap between source and detector. The redshift and increasing bandwidth are plotted in Fig. 2(b) showing very good agreement with the predictions from Eqs 4 and 7, with *a*_0,eff_ ≈ 0.25 and Δ*ω*_0_/*ω*_0_ = 0.01 (RMS). The minimum bandwidth is given by the intrinsic laser bandwidth and the asymmetric broadening due to the laser intensity. The latter also causes a small redshift (few percent) which is already visible for smallest effective divergence. Practically, this will limit the resolution of an ICS based diagnostic for LWFA applications. For the parameter set in the presented study, the broadening from the divergence dominates the spectrum for *γ σ*_*θ*,e ff_ > 0.2.

### Influence of the laser intensity

**Figure 3:**
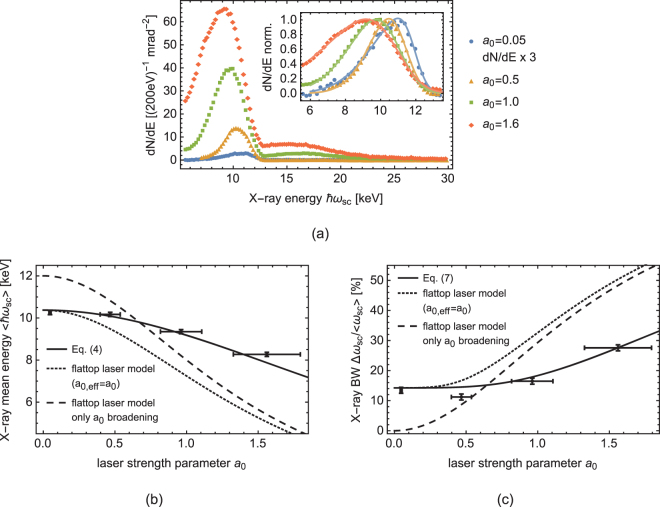
(**a**) Measured ICS spectra for various *a*_0_. The inset shows the redshift and broadening of the normalized fundamental. Dots represent measurements and solid lines are fitted skew normal distributions to derive mean energy and bandwidth. (**b**) and (**c**) Dependence of mean energy and bandwidth derived from (**a**) on the (peak) laser strength parameter *a*_0_ and comparison to Eqs 4 and 7 for the given setup (solid lines) using a Gaussian laser envelope (*a*_0,eff_ = 0.55*a*_0_). Dotted lines represent the scalings for a flattop laser model with *a*_0,eff_ = *a*_0_ and dashed lines neglect other broadening and redshifting effects from e.g. the electron beam emittance. Error bar represent the estimated, combined measurement uncertainty of *a*_0_ and the X-ray detector resolution.

In addition to the electron beam parameters, the scattering process also strongly depends on the laser parameters. Here, various effects on the scattered X-ray beam can be observed, see Fig. 3. First and foremost, increasing the laser energy leads to higher flux of scattered photons. For a laser strength parameter *a*_0_ ≈ 1, the interaction becomes nonlinear, which results in a redshift and broadening of the X-ray spectrum. These effects show the same characteristic as the emittance induced changes to the spectral shape, which can be misleading for the analysis and understanding of the interacting process. Furthermore, higher harmonics of the radiation are generated for ${a}_{0}\gtrsim 1.0$. In this work, the X-ray spectral dependence on the laser strength is investigated in the transition region from the linear to the nonlinear regime.

**Figure 4:**
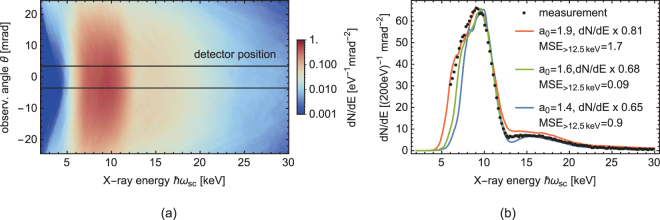
Comparison of measurement data with clara2 simulation for the *a*_0_ = 1.6 measurement. (**a**) Simulated ICS spectra in the 1/*γ* cone along the laser polarization plane (logarithmic color scale). The box indicates the detector position, for which the on-axis spectrum in (**b**) is integrated. (**b**) Comparison of the measured on-axis spectrum (black dots) with simulated spectra (solid lines). The green line shows a simulation, which matches the measurement well in shape of the second harmonic and its flux ratio to the fundamental. In red (blue), a spectrum with too high (low) laser intensity indicates the sensitivity of the second harmonic to this parameter. The mean square error (MSE) for energies above 12.5 keV is listed as an indicator for the goodness of the fit. From the comparison, the refined laser parameter for this measurement are determined as *a*_0_ = 1.6 (2.25 J and 35 fs).

Laser pulse energy and duration were measured at full power before conducting the experiment at a separate diagnostic arm. Online monitoring in the target area ensured the absence of a long term drift. However, laser beam parameters like the focal spot profile on target at full power could not be measured every shot. In order to also take into account shot-to-shot fluctuations and the influence of the laser beam transport up to the interaction point, we additionally compare the harmonics of the ICS spectrum to simulations for the highest intensity measurement. The shape of the second harmonic and the flux ratio to the fundamental radiation are very sensitive on the laser intensity and at the same time only depending weakly on other interaction parameters. Thus, they are the best suited properties of the spectrum to be used for fitting the laser parameters (see Fig. 4(b)). By this combination of laser diagnostics and simulation, the averaged laser intensity could be determined to a precision better than 15%.

The measured spectra for laser strength from *a*_0_ = 0.05 to 1.6 are shown in Fig. 3(a), normalized to a solid angle of 1 µsr per shot. The increase in X-ray flux as well as the redshift and broadening of the fundamental radiation is clearly visible. In the inset of Fig. 3(a), a skew normal distribution is fitted to the normalized measurement data up to 12 keV to determine the mean scattered energy and bandwidth of the fundamental radiation, analogous to the analysis of the previous section. The deduced bandwidth and redshift of the mean energy of the fundamental radiation are plotted in Fig. 3(b) and (c) for different laser strengths. Comparison with Eqs 4 and 7 (solid lines) shows good agreement, whereas simplified scaling laws differ significantly from the measurements. In dotted lines, the same equations are plotted, replacing the effective laser strength parameter with the (peak) laser strength parameter, resulting in a too strong scaling with the laser intensity. The dashed lines additionally neglect the broadening effects from the electron beam emittance and the intrinsic laser bandwidth, leading to a wrong prediction also for low laser intensities. For the solid lines, the spatial overlap of electron and laser beam is taken into account. The ratio of effective to peak laser strength parameter is determined to be 55%. The horizontal axes show the peak rather than effective laser strength parameter for reasons of convention and comparability. The intrinsic laser bandwidth and the effective divergence lead to the broadening and redshift in the linear regime, i.e. ${a}_{0}\ll 1$. The mean energy of the scattered spectrum drops from 10.3 keV to about 8.3 keV in the range of the covered laser power and the spectral bandwidth increases beyond 25%.

The rise of higher harmonic radiation (spectrum above 13 keV) with increasing *a*_0_ can also be seen in Fig. 3(a). For *a*_0_ = 1.6 the flux of the higher harmonics has increased to an amount that a signficant fraction of the overall scattered energy is radiated into the higher harmonics. The cut-off energy of the fundamental radiation is about 13 keV given by 4*γ*^2^*ħ* (*ω*_0_ + Δ*ω*_0_). The even harmonics of the Doppler-upshifted dipole radiation pattern of the nonlinear ICS spectrum are supposed to show only off-axis^43^. Due to the finite divergence of the electron beam in the presented setup, this pattern is however smeared out and also partially visible on axis, see Fig. 4(a).

### Comparison to 3D radiation simulations

Although scaling laws are very useful indicators, e.g. for a quick online analysis during an experiment, detailed prediction of the spectrum of an ICS source still has to rely on 3D simulations due to the high complexity of the scattering process. Implementation of scenarios as realistic as experimental conditions is useful to gain better understanding of features observed in experiments as well as to design future light sources.

Matching experimental condition for the highest laser intensity setting (*a*_0_ = 1.6) presented in the previous section, 3D radiation simulations have been carried out with the clara2 code^49^ (see Methods for further details). The interaction geometry and electron beam parameters were matched to experimental measurement quantities, including a small pointing offset of the FFS. A set of simulations have been performed varying the laser energy and pulse duration to refine the measured quantities. The resulting angle and energy resolved spectrum is plotted in Fig. 4(a). In Fig. 4(b) the on-axis spectrum (*θ* = 0) from simulation (green solid line) is compared to the measurement data (black dots). The total flux of the simulated spectrum is overestimated by only 32%, which is on the order of the measurement uncertainty e.g. from the X-ray detector’s quantum efficiency, laser power and electron bunch charge. Artifacts in the simulated spectra are caused by the sampling of the electron beam. Residual deviations in the spectral shape especially at the low energy tail of the fundamental can be explained by the approximation of the electron distribution with a Gaussian model. Further deviation might origin in the shot-to-shot fluctuations of the laser performance, which are averaged in experiment but not taken into account in simulation. Additional simulated spectra for too high and too low laser intensity (red and blue solid lines) are shown in Fig. 4(b) to indicated the sensitivity of the harmonic spectrum on the laser parameters.

From the simulation, we have estimated the total photon flux over the full emission cone for *a*_0_ = 1.6 to exceed 10^6^ photons per shot for an effective interacting charge of 1.1 pC. Similar to the effective divergence (Eq. 3) and the effective laser strength parameter (Eq. 2), the effective interacting charge is defined as the integrated charge of the ensemble of electrons, weighted with the square of the normalized local laser strength. The peak brilliance is about 10^16^ photons/(s·mm^2^·mrad^2^·0.1%BW).

## Discussion

A comprehensive experimental study on the influence of electron beam divergence and laser intensity on the shape of the ICS spectrum was presented. The good control on interaction parameters and independent tunability obtained by conventional accelerator technology was used to probe the typical parameter range of LWFA based ICS sources. Specifically, the divergence of the electron beam was varied between 0.2 mrad and 11.8 mrad and the interaction was gradually driven into the nonlinear regime by increasing the laser strength parameter up to *a*_0_ = 1.6. Additional online diagnostic on the laser pulse duration and energy, laser pointing, electron bunch charge and timing between laser and electron beam on target assured a well defined interaction of both beams. The shape of the on-axis ICS spectra was analyzed by means of the mean scattered energy and spectral bandwidth. Their quantitative dependence on electron beam divergence and laser intensity was derived from the single particle interaction equation. The obtained predictions show very good agreement with the measurement data. In contrast to existing scaling laws, the good quantitative precision opens up the possibility to reversely derive beam parameters from ICS spectra, e.g. for single shot emittance measurements of LWFA beams. For the use of ICS as a diagnostic tool in LWFA, it was found crucial to include broadening effects from both laser intensity and electron beam divergence. Both parameters have similar influence on the ICS spectrum in terms of redshift and broadening. Isolated treatment of a single parameter dependence leads to wrong conclusions due to the underlying correlations. Additionally, the geometry of the interaction in terms of spatio-temporal overlap has to be considered for correct interpretation of the measurements.

The detection limit using ICS as an online divergence diagnostic tool for parameters as presented here was well below *γ* *σ*_*θ*,eff_ = 0.23 (5 mrad, for *γ* = 46) using the quick statistics on the on-axis spectrum only. For this divergence the asymmetric broadening (Eq. 6) was larger than 5%. The resolution in divergence improves linearly with the electron beam energy, e.g. a resolution of 1 mrad is obtained for energies exceeding 100 MeV. This is sufficient to characterize LWFA electron beams, which typically show a divergence of a few milliradian at a few hundred MeV. If necessary, even higher resolution can be realized by additional comparison of the angularly resolved spectrum to simulation within the full opening cone of the scattered X-rays in a post-processing step^21,39^.

The typically higher electron energies of LWFA beams obviously require different photon detectors than used in our study. A silicon based pixel detector like used here is suitable in the photon energies up to 30 keV (electron energy up to 35 MeV), CdTe based detectors like the HEXITEC detector^50^ extend the photon energy range up to about 160 keV (electron energy up to 80 MeV). For photon energies up to a few MeV (electron energies up to a few hundred MeV), calorimetric detectors^51–54^ or magnet spectrometer based analysis of electrons resulting from Compton scattering^55,56^ can be used. Since detector development for MeV photons has seen good progress in the recent years, we are confident that a suitable detector for LWFA beam diagnostics based on ICS will be available in the near future.

In addition to the nonlinear effects on the fundamental radiation, the rise of higher harmonics has been observed for increasing laser intensities and on-axis spectra have been measured. Higher harmonics in inverse Compton scattering off relativistic electrons have been oberserved before by looking into the different spatial radiation patterns of the harmonics as spectral filtering was applied^40,41^. Very recently, Yan *et al*.^56^ performed measurement of high-order multiphoton scattering with a laser strength of up to *a*_0_ = 12. Among other findings, they were able to measure the effective laser strength during interaction through the scattered spectrum, yielding about 53% of the peak laser strength. This confirms our model to consider the interaction volume for spectral analysis (chapter 2.2). To our knowledge, spectrally resolved harmonics in the weak nonlinear regime were presented only once by Khrennikov *et al*.^57^ with an LWFA beam using an X-ray CCD camera for detection. However, no spectral correction due to potential pile-up was suggested. The correction algorithm as introduced in this article, led to a local correction of up to 25% on the higher harmonic spectrum (see Methods). This enabled us to match 3D radiation simulations to the experimentally measured spectra very accurately with the spectral shape. Comparing the shape of the second harmonic spectrum and the flux ratio of the second to the first harmonic with a set of simulations, we refined measurements of the interacting laser pulse energy and duration. This improved the precision of the quantitative comparison of the spectral shape of the fundamental spectrum to the predicted redshift and broadening. Furthermore, the overall agreement of the measured and simulated spectra supports the findings in this article.

The obtained results facilitate the use of ICS for diagnostic purposes, which could lead to an important tool towards optimizing laser wakefield accelerators for driving secondary light sources. The findings of this study also apply for the design or spectral characterization of an ICS source. In order to achieve high flux per shot, an ICS source would benefit from LWFA beams, where the interacting charge can be 2 to 3 orders of magnitude higher than in our study due to the better spatial overlap and higher initial bunch charge. Since the pulse duration of an LWFA beam is approximately two orders of magnitude shorter, a peak brilliance of at least 10^20^ photons/(s·mm^2^ ·mrad^2^·0.1%BW) seems achievable.

Potential upgrades of the X-ray source have been studied elsewhere using the same simulation tools and comparable beam parameters. A variation of the interaction scheme has been proposed to overcome spectral broadening from the laser intensity^58,59^. In the so-called travelling wave Thomson scattering scheme, the interaction length of the laser with the electron pulse is increased, which allows to operate the X-ray source at high laser energies while locally maintaining a low laser intensity during the interaction. Due to the reduced spectral bandwidth, an increase of the peak brilliance by at least one order of magnitude is expected from this scheme. Increasing the interaction length further might ultimately results in an optical free electron laser^60^.

## Methods

### Experimental setup

Electron bunches are generated with the thermionic injector of the superconducting linear accelerator ELBE and accelerated to a total energy of about 23 MeV. The beam was tightly focused to the interaction point with a dedicated final focusing system^48^. The transverse electron beam parameters including beam size, position, divergence and pointing are characterized with a 3D wire scan method (see Supplementary Material). The bunch charge on target was up to 50 pC within 20 to 100 µm RMS spot size interacting with the slightly smaller laser beam (*s* = 32 μm FWHM) from the 150 TW Ti:Sapphire laser system Draco^61^. The laser pulse parameters on target were: up to 2.25 J within 35 fs (FWHM). The laser strength was varied in experiment by adjusting the laser pulse energy *E* and keeping the pulse duration *τ* constant. The resulting laser strength parameter reads as

10 $${a}_{0}=196.95\sqrt{\frac{E[{\rm{J}}]}{{s}^{2}[{\rm{\mu }}{\rm{m}}]\cdot \tau [{\rm{fs}}]}}$$

The interaction geometry is quasi head-on with a deviation of 22 mrad (in the non-polarization plane) to clear the X-ray path for detection with an X-ray CCD camera. The arrival time of the two beam on target was optimized by means of maximum X-ray output and monitored during the experiment with a bunch arrival time monitor^62^ at a 200 fs resolution. The observed RMS timing jitter while recording a spectrum was less than 3 ps.

### X-ray detection and pile-up correction

We use a single photon counting algorithm to calculate the ICS spectrum, detected by an X-ray CCD camera (Princeton Instruments PIXIS-XO 400BR, 1340 × 400 px). The detector was pre-aligned to the electron beam axis with an alignment laser. Small corrections were applied by shadowing a metallic cross-hair with the X-rays from the ICS source. The detector covers a solid angle of 6.7 × 2 mrad^2^ at a fixed distance of 4 m to the interaction point. To cope with the strongly varying flux for the different laser and electron beam settings and to improve the signal to noise ratio, the spectra were averaged for 250 to 1500 shots. The X-ray camera has an energy resolution of 100 eV. The energy calibration is performed with the spectral lines of ^241^Am. With a pixel detector and a single photon counting algorithm for spectral characterization, there is a certain probability for pile-up, which is to falsely identify two photons hitting neighboring pixels or even the same pixel as a single photon of higher energy. This probability increases with the photon density and depends furthermore on the characteristics of the CCD chips, e.g. pixel size. Photons deposit their energy in one or more pixels, typically up to pattern sizes of 3 × 3 pixel for this specific camera and X-ray energy range. High energetic photons saturate even more pixels, but are excluded in the analysis.

The following Monte-Carlo algorithm is applied for correction of pile-up in the measured spectra (see also Supplementary Figure 3). The measured 3 × 3 patterns of detected events for each shot are normalized in energy and randomly distributed on new positions of the image. Running the same single photon counting algorithm on the so obtained synthetic images, yield the pile-up probability - the ratio of events with twice the normalized energy to the total number of events. This method inherently takes into account the fluctuations of the photon density from shot to shot as well as the increased pile-up probability due to the specific event patterns on the CCD chip. The pile-up probability determined by this method was less than 1.5% for the set of measurements with the highest photon density. Here, the ratio of incident photons to pixels on the CCD chip was 0.5%.

The correction of the spectrum was then applied on the measured data: An artificial pile-up spectrum was generated by adding the photon energy of each two randomly selected photons from the measurement data. This pile-up spectrum was scaled according to the pile-up probability and subtracted from the original spectrum. In order to conserve the radiated energy, the spectrum was finally scaled accordingly.

The correction algorithm was tested experimentally by reducing the electron bunch charge from about 50 to 10 pC and comparing the three spectra (see Supplementary Material). The reduced bunch charge leads to a weaker ICS signal at which no pile-up is expected. The pile-up corrected spectrum shows good agreement with the low charge spectrum, while the uncorrected spectrum overestimates the higher harmonics (13 to 25 keV) by 25%.

The pile-up free spectra are corrected with the transmission function from the interaction point to the detector (kapton foil, air gap, etc.) and the quantum efficiency of the silicon based detector. The X-ray background from the accelerator is measured separately and subtracted to obtain a clean ICS spectrum.

### CLARA simulations

In order to simulate the electron laser interaction and quantify the directionally resolved radiation spectra, we combined several simulations software packages^63^. The electron distribution was synthesized with 4D Gaussian distributions based on the wire scan measurement data (including any astigmatism, waist position and pointing). Laser parameters of the Gaussian laser model are based on measurement data. The interaction with a laser pulse in the paraxial approximation was simulated by including the spatially and temporally varying electromagnetic field as an external field to GPT^64^. The resulting electron trajectories were transferred to clara2^65^, a classical radiation simulation framework, which uses Liénard-Wiechert potentials in the far field approximation^66^ to predict the observable radiation flux.

The phase space distribution of the electron bunch (*Q* = 50 pC ≈ 3.1 ⋅ 10^8^ ⋅ *q*_*e*_) was sampled by *N*_sim_ = 10^4^ trajectories for radiation calculations. This sampling resolved the phase space distribution well enough to ensure stable spectral results^49^.

Due to the sparse distribution of electrons in the occupied volume *V*_*bunch*_ compared to the emitted wave length

11 $$\sqrt[3]{\frac{{q}_{e}\cdot {V}_{{\rm{bunch}}}}{Q}}\gg {\lambda }_{{\rm{sc}}},$$

the total radiation was assumed to be incoherent. Thus, the spectrally resolved energy radiated scaled with the number of electrons:

12 $${(\frac{{{\rm{d}}}^{2}I}{{\rm{d}}{\rm{\Omega }}{\rm{d}}\omega })}_{{\rm{total}}}=\sum _{i\mathrm{=1}}^{{N}_{{\rm{sim}}}}\frac{Q}{{N}_{{\rm{sim}}}{q}_{e}}\cdot {(\frac{{{\rm{d}}}^{2}I}{{\rm{d}}{\rm{\Omega }}{\rm{d}}\omega })}_{{\rm{i}}}\,,$$

with ${(\frac{{{\rm{d}}}^{2}I}{{\rm{d}}{\rm{\Omega }}{\rm{d}}\omega })}_{{\rm{i}}}$ being the spectrum of a single electron trajectory *i*. The following discretization was applied to convert the classical far-field energy contribution predicted by clara2 into number of photon per energy bin in the experiment:

13 $${N}_{\gamma }=\frac{{{\rm{d}}}^{2}I}{{\rm{d}}{\rm{\Omega }}{\rm{d}}\omega }\cdot \frac{1}{\hslash \omega }\cdot {\rm{\Delta }}{\rm{\Omega }}\cdot \frac{{\rm{\Delta }}E}{\hslash }$$

with Δ*E*=200 eV being the binning width of the histogram and ΔΩ = 10^−6^ being the solid angle of 1 mrad^2^ (200 × 200 pixels of the CCD) used to analyze the spectra in the experiment.

### Data availability

The data that support the plots within this article and other findings of this study are available from the corresponding authors upon reasonable request.

## Electronic supplementary material

Supplementary Material
