# Supplementary material for: Making spectral shape measurements in inverse Compton scattering a tool for advanced diagnostic applications
Source: Sci Rep. 2018 Jan 23;8:1398. doi: 10.1038/s41598-018-19546-0 (PMC5780516; doi:10.1038/s41598-018-19546-0)
Supplement: Supplementary file 1 — Supplementary Material [file 41598_2018_19546_MOESM1_ESM.pdf]

Making spectral shape measurements in inverse Compton scattering a tool for advanced diagnostic applications

J. M. Krämer[1,2,3,*], A. Jochmann[1,‡], M. Budde[3], M. Bussmann[1], J. P. Couperus[1,2], T. E. Cowan[1,2], A. Debus[1], A. Köhler[1,2], M. Kuntzsch[1], A. Laso García[1], U. Lehnert[1], P. Michel[1], R. Pausch[1,2], O. Zarini[1,2], U. Schramm[1,2], and A. Irman[1,+]

[1]Institute of Radiation Physics, Helmholtz-Zentrum Dresden - Rossendorf, Bautzner Landstrasse 400, 01328 Dresden, Germany
[2]Technische Universität Dresden, 01062 Dresden, Germany
[3]Danfysik A/S, Gregersensvej 8, 2630 Taastrup, Denmark
[‡]present address: National Energetics Inc., 4616 W Howard Ln, Austin, TX 78728, USA
[*]j.kraemer@hzdr.de
[+]a.irman@hzdr.de

Supplementary notes

Supplementary Note 1: Emittance measurement

The electron beam was characterized at the interaction point with a wire scan method. The horizontal and vertical profile was measured by scanning a crossed wire through the electron beam and recording the bremsstrahlung generated in forward direction with a BaF-detector. Transverse profiles were measured for different positions along the electron beam axis (z-direction) by moving the final focus system (FFS). This is equivalent to moving the crossed wire, since the beta function of the electron beam at the entrance of the FFS is several meters, whereas the applied movement was only few millimeters.

By this method, the beam waist was probed in both transverse planes, Fig. 1. Analog to a quadrupole scan, the linear beam transport function was fitted to the beam waist to obtain focal spot size and divergence. Additionally, position and pointing information were deduced by evaluating the peak positions of the beam profiles along the electron beam axis.

The normalized horizontal emittance is 20.3 ± 1.1 mm mrad with a spot size (rms) of 41 ± 1.2 μm and a divergence (rms) of 10.7 ± 0.9 mrad. The normalized vertical emittance is 18.0 ± 6.6 mm mrad with a spot size (rms) of 81 ± 2 μm and a divergence (rms) of 4.3 ± 2.0 mrad.

Supplementary Note 2: Derivation of the spectral bandwidth

Let X be a random variable that follows the distribution χ with the mean μ_χ and its standard deviation σ_χ. Let furthermore $f(\chi)$ be a transformed distribution with

$$f(X) = \frac{1}{1+X^2} \,. \tag{1}$$

The expansion of $f(X)$ as a Taylor series about the point μ_χ to first order reads as

$$f(X) \simeq \frac{1}{1+\mu_\chi^2} - \frac{2\mu_\chi(X-\mu_\chi)}{(1+\mu_\chi^2)^2} \,. \tag{2}$$

The mean of the transformed distribution $\mu_{f(\chi)}$ is

$$\mu_{f(\chi)} \simeq \frac{1}{1+\mu_\chi^2} \tag{3}$$

and its standard deviation $\sigma_{f(\chi)}$ reads as

$$\sigma_{f(\chi)} = \sqrt{\frac{1}{n}\sum_{i=1}^{n}(f(X_i)-\mu_{f(\chi)})^2} \tag{4}$$

$$\simeq \frac{2\mu_\chi}{(1+\mu_\chi^2)^2}\sqrt{\frac{1}{n}\sum_{i=1}^{n}(X_i-\mu_\chi)^2} \tag{5}$$

$$= \frac{2\mu_\chi\sigma_\chi}{(1+\mu_\chi^2)^2}\ . \tag{6}$$

The relative standard deviation accordingly results in

$$\frac{\sigma_{f(\chi)}}{\mu_{f(\chi)}} \simeq \frac{2\mu_\chi\sigma_\chi}{1+\mu_\chi^2}\ . \tag{7}$$

Applying these considerations for both the laser intensity and the electron divergence to the ICS spectrum yields the mean scattered frequency on axis in a head-on geometry of

$$\langle\omega_{\mathrm{sc}}\rangle \simeq \frac{4\gamma^2\omega_0}{1+\frac{(a_{0,\mathrm{eff}})^2}{2}+(\gamma\,\sigma_{\theta,\mathrm{eff}})^2}\ , \tag{8}$$

with $a_{0,\mathrm{eff}}$ corresponding to the mean of the laser strength parameter and $\sigma_{\theta,\mathrm{eff}}$ to the mean of the 2D interacting angle.
The contributions to the relative bandwidth of the ICS spectrum according to Eq. 7 are given by

$$\left(\frac{\Delta\omega}{\omega}\right)_{a_0} \simeq \frac{2a_{0,\mathrm{eff}}\Delta a_0}{2+a_{0,\mathrm{eff}}^2} \quad \text{and} \tag{9}$$

$$\left(\frac{\Delta\omega}{\omega}\right)_{\sigma_\theta} \simeq \frac{2\gamma^2\,\sigma_{\theta,\mathrm{eff}}\,\Delta\sigma_\theta}{1+(\gamma\sigma_{\theta,\mathrm{eff}})^2}\ , \tag{10}$$

with Δa_0 and $\Delta\sigma_\theta$ being the weighted standard deviation, defined analogously to Eq. (2) and (3) from the main manuscript and reading as

$$(\Delta a_0)^2 = \frac{\int dV\, q(x,y)\, g(x,y,ct)^2\ (a_0 g(x,y,ct)-a_{0,\mathrm{eff}}))^2}{\int dV\, q(x,y)\, g(x,y,ct)^2} \quad \text{and} \tag{11}$$

$$(\Delta\sigma_\theta)^2 = \frac{\sum_{\mathrm{i}=1}^{N_\mathrm{e}} g(x_\mathrm{i},y_\mathrm{i})^2\left(\sqrt{x_\mathrm{i}'^2+y_\mathrm{i}'^2}-\sigma_{\theta,\mathrm{eff}}\right)^2}{\sum_{\mathrm{i}=1}^{N_\mathrm{e}} g(x_\mathrm{i},y_\mathrm{i})^2}\ . \tag{12}$$

The ratio of the standard deviation to the mean for a radially symmetric 2D Gaussian function is constant as shown in supplementary note 3. This assumption for the transverse profile is reasonable for both the laser and electron beam for many applications, in particular for measurements presented in this work. Thus, the contributions to the bandwidth can be simplified as an expression of the effective laser strength parameter and effective electron beam divergence only.

$$\left(\frac{\Delta\omega}{\omega}\right)_{a_0} \simeq \frac{2C_{\mathrm{RSD}}\,a_{0,\mathrm{eff}}^2}{2+a_{0,\mathrm{eff}}^2} \quad \text{with} \quad C_{\mathrm{RSD}} \simeq \begin{cases} 0.44 & ,\ \text{for } r_l \ll r_e \\ 0.36 & ,\ \text{for } r_l = r_e \\ 0.25 & ,\ \text{for } r_l \gg r_e \end{cases} \tag{13}$$

$$\left(\frac{\Delta\omega}{\omega}\right)_{\sigma_\theta} \simeq \frac{1.05\,(\gamma\sigma_{\theta,\mathrm{eff}})^2}{1+(\gamma\sigma_{\theta,\mathrm{eff}})^2} \tag{14}$$

In total, the relative bandwidth of the ICS spectrum sums up to

$$\frac{\Delta\omega_{\mathrm{sc}}}{\omega_{\mathrm{sc}}} \simeq \sqrt{\left(\frac{\Delta\omega_0}{\omega_0}\right)^2+\left(\frac{2\Delta\gamma}{\gamma}\right)^2+\left(\frac{\Delta\omega}{\omega}\right)_{\sigma_\theta}^2+\left(\frac{\Delta\omega}{\omega}\right)_{a_0}^2}\ . \tag{15}$$

Supplementary Note 3: Ratio of SD to mean for a 2D Gaussian distribution

A radially symmetric 2D Gaussian distribution in polar coordinates is given by

$$g(r) = \frac{1}{2\pi\sigma^2} e^{-\frac{r^2}{2\sigma^2}} \ . \tag{16}$$

For r being distributed according to $g(r)$ the expectation (mean) μ_r is calculated as

$$\mu_r = \int_{-\pi}^{\pi}\int_{-\infty}^{\infty} r g(r) r dr d\phi \tag{17}$$

$$= \int_{-\pi}^{\pi}\int_{-\infty}^{\infty} \frac{r^2}{2\pi\sigma^2} e^{-\frac{r^2}{2\sigma^2}} dr d\phi \tag{18}$$

$$= \sqrt{\frac{\pi}{2}}\sigma \ , \tag{19}$$

the standard deviation σ_r is

$$\sigma_r^2 = \int_{-\pi}^{\pi}\int_{-\infty}^{\infty} (r-\mu_r)^2 g(r) r dr d\phi \tag{20}$$

$$= \int_{-\pi}^{\pi}\int_{-\infty}^{\infty} \frac{(r-\sqrt{\pi/2}\sigma)^2 \cdot r}{2\pi\sigma^2} e^{-\frac{r^2}{2\sigma^2}} dr d\phi \tag{21}$$

$$= \left(2 - \frac{\pi}{2}\right)\sigma^2 \tag{22}$$

$$\sigma_r = \sqrt{2 - \frac{\pi}{2}}\sigma \tag{23}$$

and the relative standard distribution reads as

$$\frac{\sigma_r}{\mu_r} = \sqrt{\frac{4}{\pi} - 1} \approx 0.523 \ . \tag{24}$$

For non radially symmetric 2D Gaussian distributions with $\sigma_x \neq \sigma_y$, the relative standard distribution is larger and can be as large as 0.756.

Supplementary figures

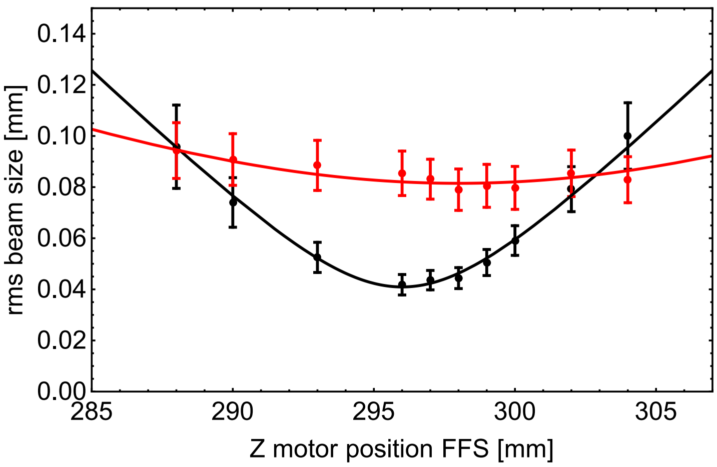

Supplementary Figure 1. Emittance measurement with a wire scan method for horizontal (black) and vertical (red) plane. The beam size at various positions along the waist is obtained by scanning a wire through the beam (dots). From the fit (solid line), spot size, divergence and emittance is determined.

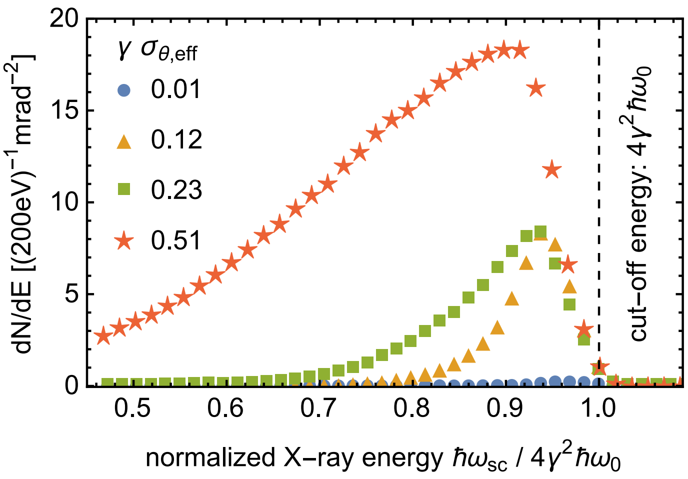

Supplementary Figure 2. Spectra from Fig. 2(a) in the main manuscript but with absolute photon numbers per shot. For $\gamma\sigma_{\theta,\mathrm{eff}} = 0.51$ the electron beam was focused the most yielding the best spatial overlap, highest photon flux and largest bandwidth. The interacting charge is estimated to about 90 fC. $\gamma\sigma_{\theta,\mathrm{eff}} = 0.23$ and 0.12 show the same peak flux while the bandwidth was reduced for $\gamma\sigma_{\theta,\mathrm{eff}} = 0.12$, where the electron beam was diverging at interaction point. The interacting charge is estimated to about 23 fC for interaction at the focus position and about 6 fC for the diverging beam. For the case without final focusing ($\gamma\sigma_{\theta,\mathrm{eff}} = 0.01$) the overlap was very poor (interacting charge < 0.1 fC and the photon yield very small, accordingly.

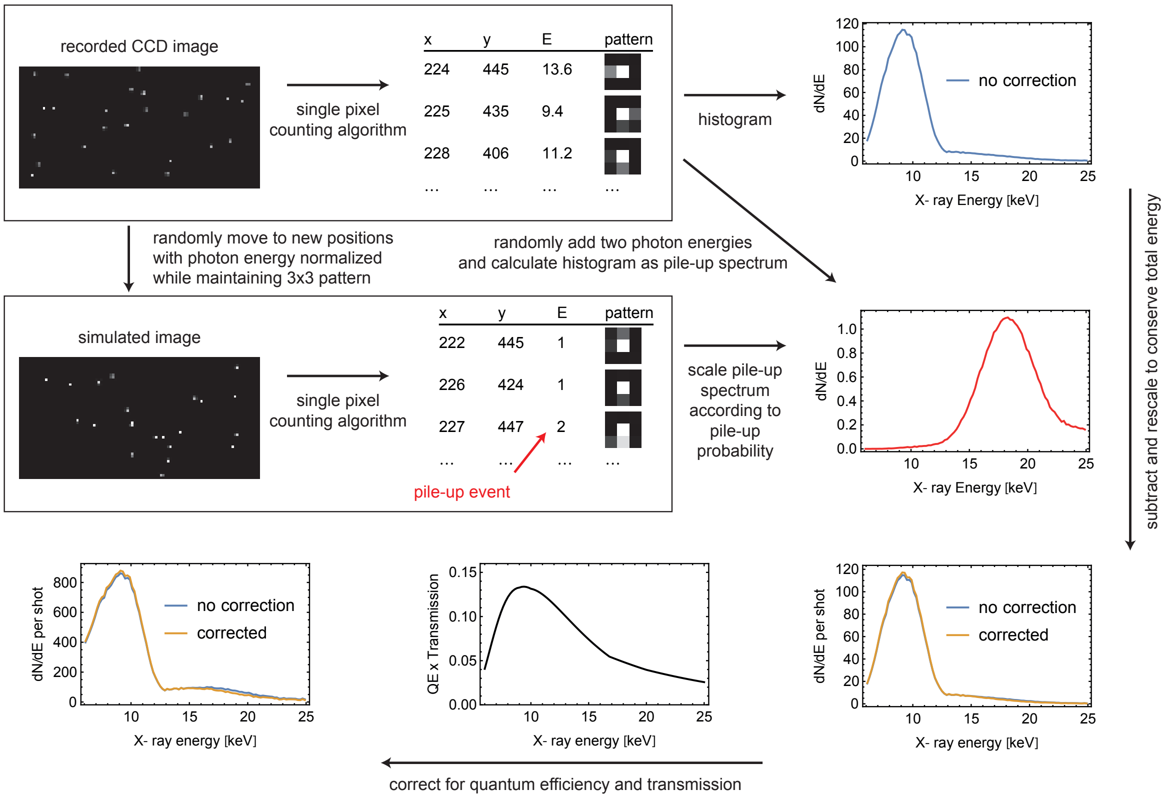

Supplementary Figure 3. Schematic figure of the pile-up correction algorithm as described in the Method section of the manuscript.

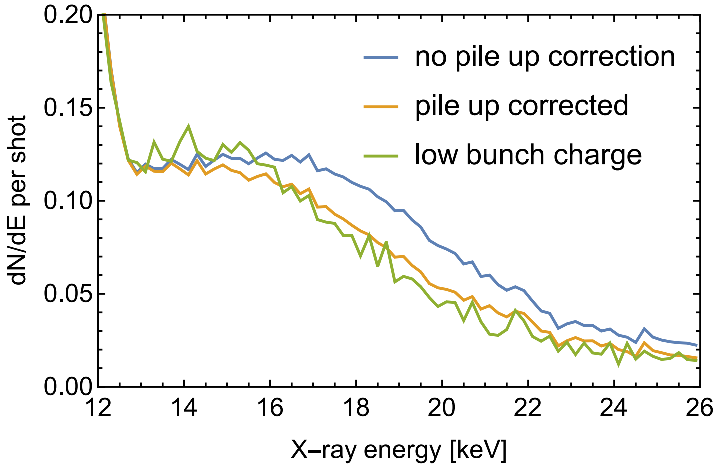

Supplementary Figure 4. Pile-up correction of higher harmonic spectrum. The difference in the spectra from low and high bunch charge measurements is caused by pile-up. The correction algorithm applied on the high bunch charge measurement results in the undiluted higher harmonic spectrum.

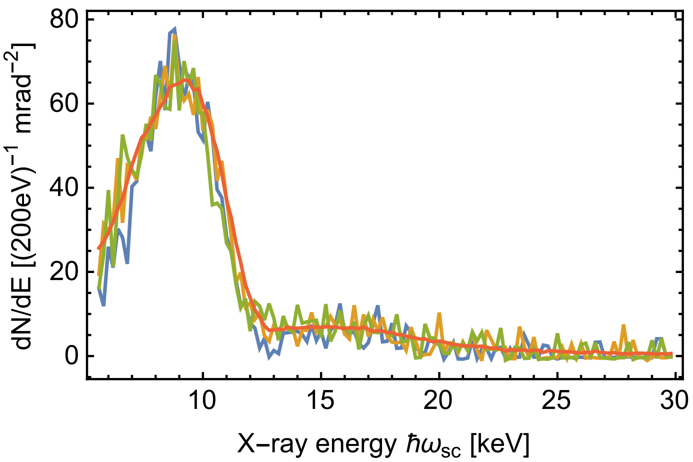

Supplementary Figure 5. Three exemplary consecutive single shot spectra (blue, green, yellow) for the $a_0 = 1.6$ measurement as presented in Fig. 3(a). The red curve is a copy of the averaged spectra from the main manuscript. The detected photon flux on the camera was optimized for this setting while keeping the pile-up probability at a moderate level of 1.5%.
